# Fiber Orientation and Concentration in an Injection-Molded Ethylene-Propylene Copolymer Reinforced by Hemp

**DOI:** 10.3390/polym12122771

**Published:** 2020-11-24

**Authors:** Antoine Dupuis, Jean-Jacques Pesce, Paulo Ferreira, Gilles Régnier

**Affiliations:** 1 PIMM, Arts et Métiers Institute of Technology, CNRS, Cnam, HESAM Université, 151 Boulevard de l’Hôpital, 75013 Paris, France; antoine.dupuis@ensam.eu (A.D.); paulo.ferreira@ensam.eu (P.F.); 2 Faurecia Interior Systems, 8 Rue Emile Zola, 60110 Méru, France; jean-jacques.pesce@faurecia.com

**Keywords:** natural fiber, fiber reinforced polymers, injection molding, fiber orientation, thermo-elastic properties, fiber elastic anisotropy

## Abstract

This paper characterizes and analyzes the microstructures of injection-molded polypropylene parts reinforced with 20 wt% of hemp fibers in order to understand the process induced variations in thermomechanical properties. In-thickness fiber orientation and fiber content were determined by X-ray tomography along the flow. The fiber content along the flow path was also determined by direct fiber content measurements after matrix dissolution, showing an increase of 2%/100 mm for a 2.2 mm-thick plate due to fiber migration during the filling stage. A typical shell/core structure for fiber orientation in injection molding was observed, but with a very clear transition between the layer solidified under high shear rates and the core in which the fiber content was reduced by more than 50%. The orientation of hemp fibers is lower than the one of glass fibers, especially in thickness direction. However, the overall fiber orientation in the injection direction induces significant anisotropic thermomechanical properties, which cannot be explained by simple micromechanical models that consider isotropic mechanical properties for hemp fibers. These phenomena must be taken into account in process simulation codes for injection molding to better predict thermomechanical properties as well as part shrinkage and warpage to design molds.

## 1. Introduction

In the automotive and transportation industry, a reduction in the weight of vehicles is essential for both pollution and safety. To address this issue, the use of natural fibers as reinforcements for polymer composites has received a growing interest in the last decades [1,2]. Natural fibers have attractive physical properties compared with traditional synthetic fibers such as glass or carbon fibers because of their low density and high specific mechanical properties [3,4,5]. In addition, these fibers have two main environmental assets: Their low energy demand with local production and their renewability [6]. For instance, polypropylene (PP) reinforced with hemp fibers can reach a specific stiffness equivalent to PP reinforced with glass fibers and can reduce the weight of some interior automotive parts such as doors or instrument panels by up to 25% when compared to PP reinforced with 20 wt% talc. These bio-sourced composites are particularly used in the automotive industry for injection molding, which is one of the most flexible, reliable, and cost-effective manufacturing technologies for plastic components with complex geometry [7].

However, natural fibers have large geometrical variations (microfibril angles and embranchments, high tortuosity, aspect ratio) as well as biochemical compositions (cellulose, lignin contents), which substantially affect the mechanical behavior of the composite. These variations, which impact the binder between single fibers in the bundles, are induced by fiber maturity, harvesting time, extraction and production methods, and treatment and storage procedures [5].

Regardless of the type of fibers used in the composite, local variations in fiber orientation determine the variation in the thermomechanical and dimensional properties of injection-molded thermoplastic composites [8]. These variations are attributed to the flow-induced orientation during the filling of the mold cavity. It is therefore of prime importance to control the fiber orientation during the molding process by accurately describing the flow behavior of the suspension during the filling stage [9]. For this purpose, the in-thickness fiber orientation of injected parts has already been substantially analyzed in the literature [10,11,12,13,14]. For each part, several layers were observed with a specific fiber orientation. A skin/core structure is typically found, specific to the transient, non-Newtonian, and non-isothermal flow experienced in injection molding. Fiber orientation is governed by fountain flow effects during mold filling [15]. An extensional flow occurs at the advancing flow front and results in fibers oriented in the flow direction in the shell layer, whereas fibers in the core region, being subject to a very low shear gradient, are more preferentially oriented transversely to the flow [16].

The microstructure of such composites is also affected by a significant segregation of fillers resulting from the molding process. One of the first studies related to fillers segregation is the work of Kubat and Szalanczi [17]. The authors found an increase in glass spheres content with longitudinal direction which is reinforced by the diameter of the glass spheres and the flow length. A decade later, Hegler and Mannig studied the segregation of glass fibers in styrene-acrylonitrile copolymer (SAN) and polyamide 6 matrix [18]. Compared to glass-bead-filled thermoplastics, a homogeneous distribution of fibers along the flow length is found especially for short-fiber-filled system. This statement was confirmed by Toll and Andersson, who noticed an evenly-distributed fiber content for short glass fiber-filled polyamide 66, with an aspect ratio of 60 [19]. A segregation of particles towards the core layer was also noticed, which is mainly attributed to fountain flow effects [20]. More recently, Ramzy et al. observed a linear increase in natural fiber content, as hemp, along the flow length [21]. An interesting feature, not encountered for glass fillers, is the building of fiber clusters which easily flow with the injection path.

As described above, it is necessary to control many process parameters, which have a direct impact on the material microstructure, in order to ensure the mechanical properties and dimensional accuracy required in automotive industry applications for component assembly. For injection molding processes, many researchers report that the main injection parameters affect the overall shrinkage: Cooling time, packing pressure [22,23], melt temperature [24], and injection flow rate [25]. Indeed, local anisotropy in the microstructure, induced by the injection parameters, leads to shrinkage heterogeneity, which can consequently induce warpage in the slender parts.

In this context, a specific mold was designed to manufacture PP plates reinforced with hemp fibers. First, the present paper analyzes the effects of the process parameters such as flow rate, packing pressure, and gate configuration on the induced microstructure. Then, thermomechanical properties and shrinkage measurements are assessed to understand the links between the induced microstructure and these end-use properties.

## 2. Experimental

### 2.1. Processed Material

The thermoplastic matrix used in this study consists of a heterophasic ethylene-propylene copolymer. The Young modulus at 23 °C was measured at 1450 MPa, the density at 905 kg/m^3^ and the fusion temperature at 163 °C.

**Figure 1:**
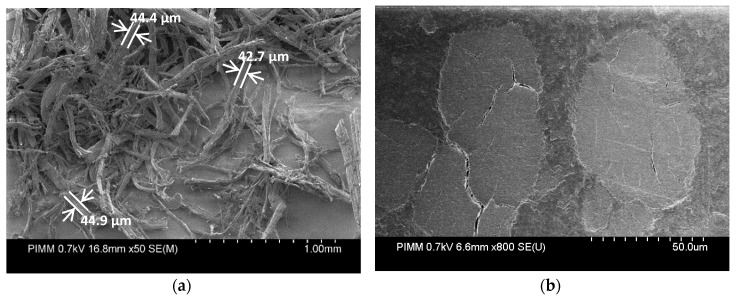
Scanning electron microscope images of hemp fibers (**a**) before mixing with the polymer matrix and (**b**) in an injected biocomposite sample.

Hemp fibers (Cannabis sativa L., variety Fedora 17) were supplied by Fibres Recherche Développement (FRD, Troyes, France) and used as reinforcement. The experimental composite material was supplied by Automotive Performance Material (APM, Fontaine-lès-Dijon, France) in the form of solid pellets for the injection molding process. The degree of retting was adjusted by the supplier for typical use in common composite applications. Hemp fibers have an average length of 600 µm and an average aspect ratio of 20. However, as for most natural fibers, these values may be highly variable: The aspect ratio can reach up to 100 for single detached fibers (Figure 1). The weight fiber content of pellets was measured by matrix dissolution and found to be 22%. The biochemical composition of hemp fibers can be found in the work of Puech et al., characterized according to the Van Soest method [26].

### 2.2. Injection Molding and Preparation of Samples

**Figure 2:**
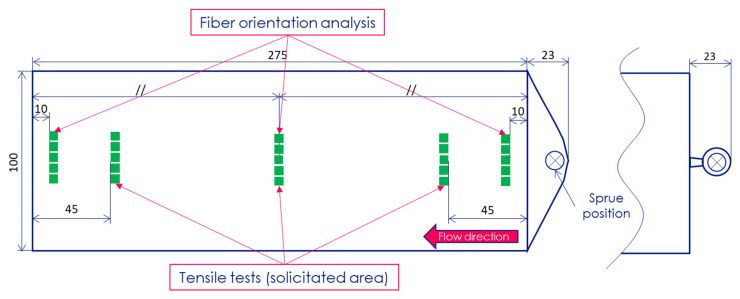
Positions and injection configurations of the part samples used in the study: (Left) fan gate, (right) tab gate.

A specific plate mold equipped with a hot runner was designed and mounted on a DK Codim injection molding machine (DK Technologies, Gonesse, France), with a clamp force of 1750 kN and a screw diameter of 36 mm. Rectangular injected plates measuring 275 × 100 × 2.2 mm^3^ were molded with one of two feeding systems: A fan gate or a tap gate (Figure 2). Nozzle temperature was set at 185 °C and ram speed at 30 and 45 mm/s, which gave a filling time of 2.4 and 1.6 s, respectively. Pressure at switchover was about 900 bars. For samples with a holding pressure phase, a constant pressure of 450 bars was maintained for 10 s. The cooling phase, which started after the holding pressure phase, lasted 25 s with a regulation temperature for the mold fixed at 30 °C. The different injection parameters considered in the study are listed in Table 1.

Samples of 5 × 5 × 2.2 mm^3^ were cut at five specific locations for microstructure characterizations (Figure 2). For fiber orientation characterizations by X-ray tomography analysis, the samples were only cut at three locations, which were subjected to different kinds of flow fields and named according to the work of Bay and Tucker [27]: Entry (10 mm from the injection gate), lubrication (137.5 mm), and near-end-of-fill (265 mm).

Another aim of the study was to determine the persistent effect of the gate configuration on the fiber orientation distribution throughout the thickness. Indeed, the geometry and thickness of the fan gate configuration was studied to ensure a linear flow front from the cavity entry. For the tab gate, a radial flow front begins at the entry and tends to become linear in the middle of the cavity.

### 2.3. Fiber Content Measurement

The protocol used for natural fibers in the biocomposite is more complex than that used for mineral fibers in which the polymer matrix can be removed by pyrolysis. Samples with a mass of around 1 g were first weighted. The dissolution of the PP matrix was achieved for 3 h at a high temperature (170 °C) using 1,2,4-trichlorobenzene. After the complete dissolution of the PP matrix, a vacuum filtration process was performed using a Büchner system. Hemp fibers were then weighted after drying for 12 h. The fiber concentration corresponds to the ratio between the weight of the hemp fibers and the initial weight of the samples.

### 2.4. X-ray Microtomography Scans

X-ray microtomography characterization was conducted by the Navier laboratory (ENPC, Champs-sur-Marne, France). The platform comprises an “Ultratom” microtomography designed by RX-Solutions. A Hamamatsu L10801 micro-focal source (230 kV, 200 W, 5 µm) was used for scan acquisition with the following parameters: X-ray tube voltage 70 kV, X-ray tube current 70 μA, a spot size of 5 µm, and a voxel size of 5.45 µm. Volumes of 5 × 5 × 2.2 mm^3^ were scanned for 24 cases. A stack scan was used for sample acquisition, with a rotation mirror of 90°.

Due to the high degree of tortuosity in hemp fibers and their variable aspect ratio, techniques based on single fiber segmentation or segmentation between the fibers and the matrix followed by a skeletonization algorithm cannot be used. Indeed, for such characterization techniques, fibers must be unidirectional and resolved by the acquisition.

Computed tomography data were analyzed using VG Studio Max 3.1 software from Volume Graphics (Heidelberg, Germany). As in the study of Baradi et al. [28], fiber orientation and volume fraction were evaluated using a local filtering method, which has a gradient threshold of gray values to dissociate hemp fibers from the thermoplastic matrix and computes the local fiber orientation for each voxel. A regular hexahedral mesh of the area of interest was used to average the computed voxel values inside each mesh element. The mesh element size was fixed at 0.11 mm in the injection plane (X and Y) and at 0.7 mm in the thickness direction (Z). About 50,000 elements per sample were then analyzed. For each mesh element, VG Studio Max 3.1 computed the second-order fiber orientation tensors, as defined in [29], and a fiber content.

The mesh for microstructure reconstruction was defined in such a way that the three main fiber orientation tensor components broadly correspond to the directions of the Cartesian coordinates: a_xx_ component corresponds to the fiber orientation in X direction (flow direction), a_yy_ in Y direction (transverse to flow), and a_zz_ in Z direction (thickness direction).

### 2.5. Ultrasonic Wave Measurements

Microstructural anisotropy is also achievable using ultrasonic measurements. The procedure has already been used to characterize specific microstructures [30,31,32] or assess damage evolution [33] inside reinforced materials. For this study, ultrasonic waves are a simple way to obtain a direct image of the distribution of the in-plane fiber orientation through the evolution of shear wave velocity, which is proportional to the shear modulus in the shear direction.

The ultrasonic apparatus is composed of two probes: The first transmits transverse waves with a 5 MHz frequency, and the second, separated by a constant distance of 500 mm, receives them. The diameter of the probe is 10 mm. The sample is placed between the two probes at an angle of about 55° from the incident ultrasonic waves to maximize shear wave velocity, with the propagation direction inside the material being determined by the Snell-Descarte law [34].

Consequently, the material undergoes ultrasonic shear stress in the plane defined by the incident wave and the vector normal to the plane of the sample. As explained by Shirinbayan et al. using the same experimental apparatus, two limits are experienced in these testing conditions [32]. First, the majority of fibers are found in the ultrasonic shear stress plane. This configuration corresponds to the maximum section of fibers subjected to shear stress, which increases the velocity. Consequently, for longitudinally oriented fibers, the measured value of shear wave velocity, *V_SW_*, will be maximal. Nevertheless, in the case of transverse orientation, the sheared section of the fibers is reduced, leading to a minimal value of shear wave velocity. For all the samples analyzed with this measurement method, a 360° rotation was performed in increments of 30°, while the maximal amplitude of the ultrasonic signal was measured and recorded at each point. Data processing determines the in-plane components of the second-order orientation tensor. Shear wave velocities in neat PP and biocomposite samples were compared to evaluate the effect of hemp fibers on wave transmission velocity.

### 2.6. Young’s Modulus Measurement

**Figure 3:**
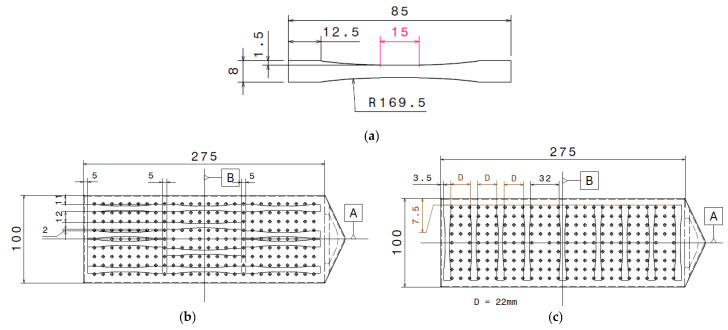
(**a**) Tensile sample shape and dimensions as well as localizations of tensile samples in the (**b**) longitudinal and (**c**) transverse directions.

To measure Young’s modulus in the samples, three areas were selected (Figure 3). Quasi-static tensile tests were carried out on the biocomposite using an Instron 5581 machine (Instron, Norwood, MA, USA) with a mechanical extensometer. The crosshead displacement speed was fixed to 0.6 mm/min, corresponding to a deformation rate close to 1%/min according to ISO 527-2 standard. A specific shape of tensile samples was chosen to maximize the high stress area and minimize the transition between this area and the part in the grip, thus avoiding stress concentration and breakage in this transition (Figure 3a). For each part area (i.e., entry, lubrication, and near-end-of-fill regions) and each direction (i.e., longitudinal and transverse), samples were cut from two injected plates by water jet cutting.

### 2.7. Dilatometric Properties

A dynamic mechanical analysis device (DMA-Q800, TA instrument, New Castle, DE, USA) was used to measure the linear thermal expansion coefficients of samples. The heating/cooling rate was set at 3 °C/min in the temperature range of 10–120 °C under nitrogen. The method consists of applying a constant preload force of 0.5 N in tensile mode to samples measuring 30 × 5 × 2.2 mm^3^. The strain evolution is measured during the imposed temperature cycle. The coefficient of thermal expansion (CTE) was determined during the cooling stage, as residual stress is relaxed during the heating ramp, inducing an apparent non-linear and irreversible deformation with temperature.

### 2.8. Shrinkage Measurement Method

In-plane shrinkage measurements were taken using a DEKTAK profilometer (Bruker, Billerica, MA, USA). Specific 2 mm square engravings, made by electro-discharge machining and corresponding to an approximate extra thickness of 50 μm on the injected parts, were placed in the three different regions considered in this study (i.e., entry, lubrication, and near-end-of-fill). Each engraving was separated from other engravings by a reference distance of 10 mm in longitudinal and transverse directions in the mold cavity. The radius of the stylet used for the measurements was 1.5 μm. A Python script was used to detect the slots of each marker and then evaluate the distance between two markers.

## 3. Results and Discussion

### 3.1. Fiber Concentration along the Flow Path

To assess the changes in filler concentration in the injected part, density and direct fiber content measurements were tested. These experimental measurements primarily aim to determine a relation between the distance of the samples taken from the injection gate and a change in the density or fiber content.

**Figure 4:**
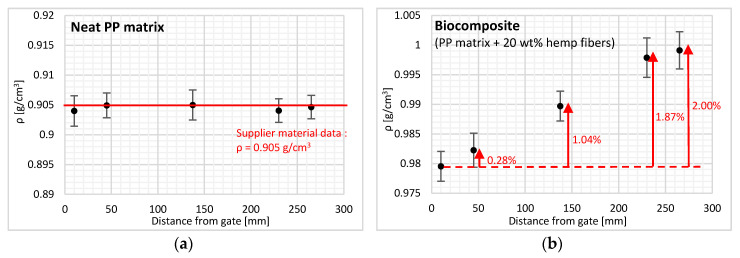
Density along the flow path for the (**a**) neat polypropylene (PP) matrix and (**b**) PP reinforced with hemp fibers. Error bars correspond to plus or minus the standard deviation.

First, density measurements were performed on the injected parts of neat PP. Samples sized 5 × 5 mm² were cut with a saw and deburred, respecting the locations uncovered in the experimental part. Figure 4 below shows that the variation in density depending on the distance from the injection gate is non-significant. Moreover, all the measured values are similar to the density given by the supplier (ρ = 0.905 g/cm^3^), thus confirming the absence of density variation in the injected part of neat PP. These results confirm that flow length has no influence on polymer crystallinity as confirmed by differential scanning calorimetry, which showed that the melting enthalpy remained constant, regardless of where the samples were cut on the injected part.

**Figure 5:**
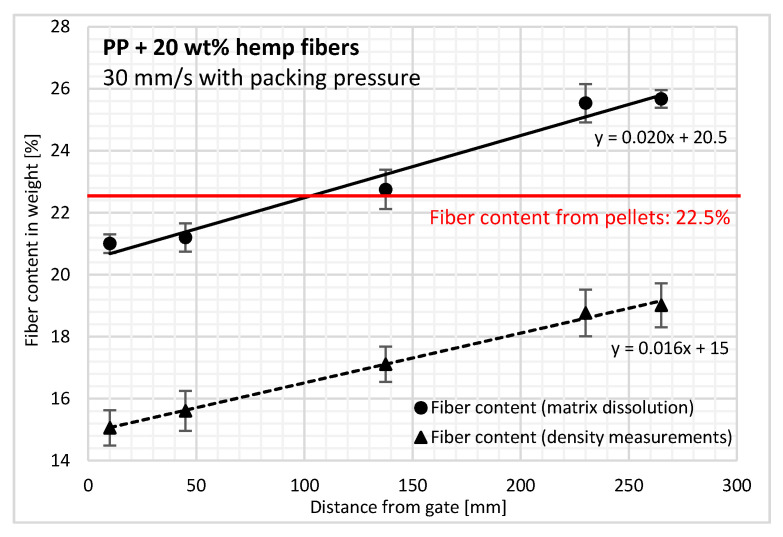
Hemp fiber content changes along the flow path for PP reinforced with hemp fibers using the fiber extraction method and density measurements. Error bars correspond to plus or minus the standard deviation.

For measurements performed on biocomposite material, the results show a density increase of up to 2% with flow length at 265 mm (near-end-of-fill region). This linear increase in density is clearly the consequence of changes in the filler content with flow length. A fiber content is determined from neat PP density (Figure 5). Probably due to the irregular dispersion of the hemp fibers and of inner porosities, the overall standard deviation of biocomposite density measurements is slightly higher (0.0032 g/cm^3^) than the one of PP matrix measurements (0.0025 g/cm^3^).

A direct fiber concentration measurement method was also used to verify the trend observed for the density results. The fiber content also increased linearly with the distance from the injection gate (Figure 5), that is, from 21% at the cavity entry (10 mm from the gate) to approximately 26% in the near-end-of-fill region (265 mm). This increase is in accordance with the trend previously observed by Ramzy et al. who studied the behavior of PP reinforced with natural hemp fibers in a spiral flow mold [21]. For fillers of different types, Kubat and Szalanczi as well as Danès et al. found a minimum close to the gate and a pronounced maximum at the opposite mold wall with glass spheres and aluminum fibers, respectively [17,35].

Comparing the results from the density and fiber content measurements, we observe that the slopes are similar for both methods and correspond to an increase of 5% for the entire length of the part, that is, 2%/100 mm flow.

However, the absolute values for each method are separated by a gap of around 6%, which needs to be clarified. On the one hand, density values are underestimated by the presence of inner porosities in the samples. The incorporation of hemp fibers in the PP matrix with their specific shape and composition results in a higher porosity content, especially at the fiber/polymer interface and inside the bundles of fibers. Moreover, inherent to the manufacturing process, as injection molding is applied at high screw speed, air entrapment occurs, leading to the inclusion of air during processing [5]. On the other hand, there is a risk that some matrix or additives remain after the matrix dissolution process, thus overestimating the fiber weighting. These phenomena explain the constant gap observed between the results of each method. We can therefore conclude that the porosity content is less than 6% of the injection-molded PP reinforced with hemp fibers considering a well-admitted fiber density value of 1.4 g/cm^3^. Nevertheless, this density is very difficult to measure. Moreover, a certain biological dispersion may exist on this value and consequently on the porosity content, knowing that a density of 1.35 g/cm^3^ would induce a level of about 3% of porosity.

### 3.2. In-Thickness Fiber Morphology

**Figure 6:**
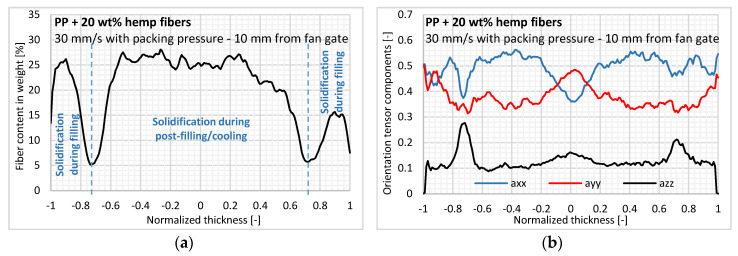
At the cavity entry region, measurement of (**a**) fiber content and (**b**) three components of the second-order orientation tensor inside the sample normalized thickness (a_xx_: Flow; a_yy_: Cross-flow; a_zz_: Thickness).

Figure 6a shows the variation of in-thickness fiber content determined by X-ray tomography. We can first observe a very heterogeneous in-thickness fiber content characterized by two symmetrical layers with a very low fiber content (<10% in weight) and located approximately 0.3 mm from the mold wall. These layers would correspond to the transition between the layers solidified during mold filling and the layers solidified during post-filling/cooling. A numerical simulation of the industrial code performed in the same injection conditions shows a very good correlation between the estimated in-thickness solidification time and the position of the experimental transition layers for the three sample positions studied here. Thus, depending on the fiber content, the three symmetrical layers can be considered in terms of sample thickness, namely:

- Skin: Instantaneously solidified upon contact with the cold mold wall and estimated to be 0.1 mm thick.
- Transient layer: Subjected to very high shear rates, causing a gradual demixing of the fibers as the layer solidifies, and characterized by a very low fiber content (<10% in weight).
- Core: Subjected to low shear rates during mold filling and to solidifying during post-filling/cooling.

Shear-induced fiber concentration, meaning a fiber content gradient inside the part thickness, was also observed by [14,36] for a steady-state Couette shear flow with nylon rods. This phenomenon is barely identified for PP reinforced with glass fibers in which fiber content only slightly differs [12] or even remains constant [37]. The phenomenon of fiber demixing in layers subjected to very high shear rates was also observed in powder injection molding by [38]. In our view, the tortuosity and high flexibility of the hemp fibers reinforce the shear-induced fiber migration, while the extracted fibers in this layer are carried further in the flow, thus explaining the increase in fiber content with the flow.

Regarding the in-thickness fiber orientation, the off-diagonal tensor components had very low values, indicating that the plate reference axes X, Y, and Z are very close to the main principle directions. In Figure 6b, we can observe the low overall orientation compared to the more conventional short reinforcement fibers such as glass [8]. A relatively high orientation level in the thickness direction can be identified, ranging from 0.1 to 0.15. Both aspects on the hemp fiber orientation can be explained by the low density difference with the PP matrix (1.4 g/cm^3^ and 0.905 g/cm^3^, respectively) as well as the flexibility of these fibers, as they have a relatively high aspect ratio and a high degree of tortuosity. Coupled with the low lignin content of hemp fibers (≤2%), the bundles are soft and less cohesive, thus reducing their interaction with the velocity or shear rate changes and their orientation ability [39].

In terms of the results for in-thickness fiber morphology, an isotropic orientation state is observed in low fiber content layers, with a sudden increase in thickness orientation. Based on our knowledge, this result seems paradoxical, since the high shear rates applied to these layers during mold filling would induce a high orientation level.

Lastly, a typical shell/core structure is observed for the fiber orientation (Figure 6b). At the core layer, fibers are oriented transversely to the injection direction due to the extensional flow at the flow front, whereas fibers are oriented more in the flow direction in the shell layers, subjected to shear flow during mold filling. In skin layers, instantly solidifying upon mold contact, a random in-plane orientation is identified, which results from the flow fountain. The in-thickness fiber orientation for injection-molded parts has already been analyzed extensively in the literature [10,11,12,13,14], showing a more pronounced shell/core structure for conventional fibers like glass and carbon.

#### 3.2.1. Effect of Injection Flow Rate

**Figure 7:**
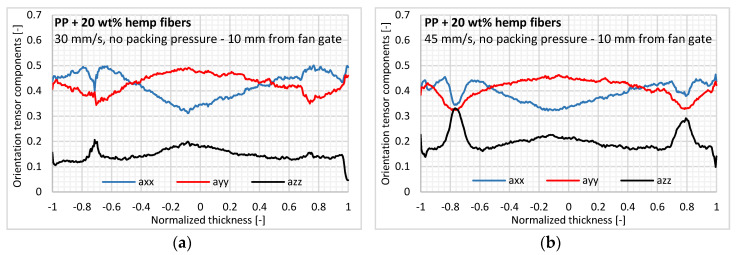
In-thickness fiber orientation for two injection ram speeds: (**a**) 30 and (**b**) 45 mm/s.

In this study, two injection ram speeds were considered: 30 and 45 mm/s. A flatter orientation profile can be observed for the injection ram speed of 45 mm/s compared to 30 mm/s, which induces less orientation at a higher flow rate (Figure 7). Even if the difference between both profiles is minimal, this result also seems paradoxical because higher shear rates applied at 45 mm/s would orient the fibers more in the injection direction. However, according to [11], applying higher shear rates would induce a flatter velocity profile for the shear-thinning polymer with less reorientation ability in the injection direction. Moreover, a significantly higher orientation in the thickness direction at 45 mm/s is observed with average values of 0.151 (at 30 mm/s) and 0.192 (at 45 mm/s) for the second-order orientation tensor component.

**Figure 8:**
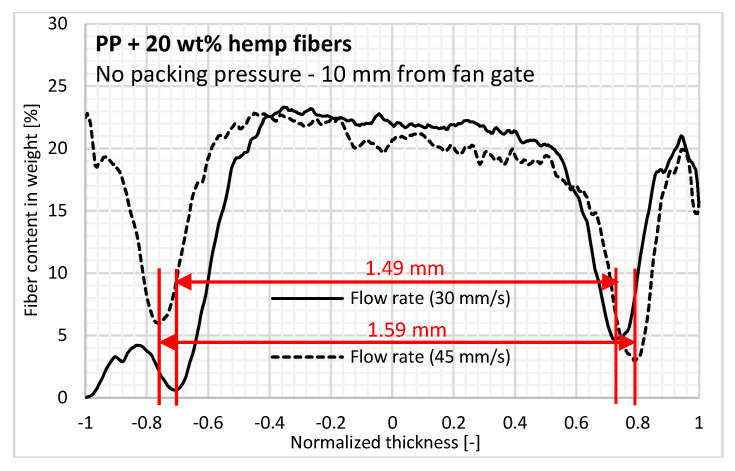
Effect of injection flow rate on in-thickness fiber content3.6.2. Effect of Packing Pressure.

A thicker core layer with transversely oriented fibers is also barely identified at a high flow rate without packing pressure. Indeed, a higher injection velocity reduces the thickness of the solidified layer during filling. The skin layer thickness is reduced by a consistent value of 50 µm (2.3% of the entire thickness) at each side of the sample at a high flow rate (Figure 8). Then, after filling the molten polymer, the flow channel is enlarged in the cavity, thus inducing a larger core layer with transverse orientation [16].

#### 3.2.2. Effect of Packing Pressure

**Figure 9:**
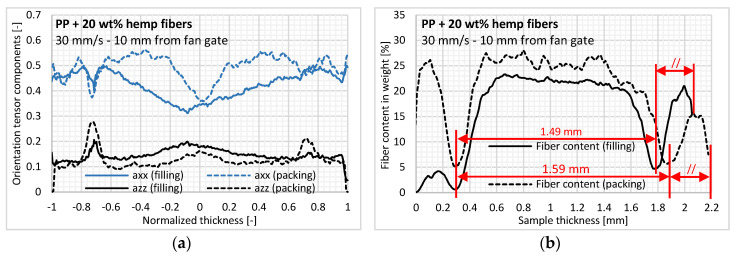
Effect of packing pressure on (**a**) fiber orientation state and (**b**) in-thickness fiber content.

The effect of packing pressure is analyzed by considering the samples injected without packing pressure and those injected with a holding pressure of 400 bars applied in the nozzle for 10 s. The impact of packing pressure is reduced to the layers located at the thickness core and is even more visible close to the injection gate, where the effect of the packing pressure is the greatest (Figure 9a). We observe that the application of packing pressure at the end of filling leads to a reduction in the core layer thickness (Figure 9a), thus implying an increase in fiber orientation in the flow direction [14,40]. The transition between shell and core layers is more distinct and clearly visible compared to the injection without packing pressure; it is subject to a mixture of shear and extensional flows [41].

The distance from the mold wall to the low fiber content peak did not change with the application of packing pressure (Figure 9b), which confirms that these layers solidify during the filling stage. Thus, packing pressure only induces an enlargement of the central layer estimated at 100 µm (4.5% of the entire sample thickness) (Figure 9b), consistent with the variation in plate thickness of 120 µm.

#### 3.2.3. Effect of Flow Length

**Figure 10:**
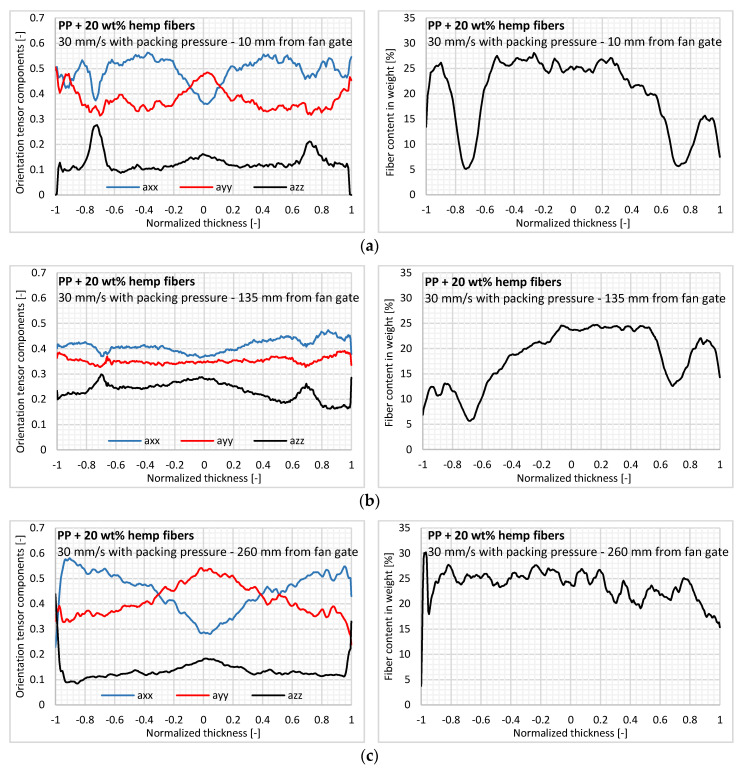
In-thickness fiber orientation and fiber content for the three regions of the injected part: (**a**) Entry, (**b**) lubrication, and (**c**) near-end-of-fill.

First, we can observe an influence of flow length on the evolution of in-thickness fiber content. Indeed, close to the injection gate, a steep decrease in fiber content is observed from 25% to 5% in weight (Figure 10). This transition is still visible in the lubrication region but with a lower fiber content difference (around 10%) (Figure 10b). In the near-end-of-fill region, there is no transition layer, and the high fiber content remains constant across the thickness (Figure 10c). We can deduce that the effect of flow length on fiber demixing is reduced along the flow path. Moreover, the further away from the injection gate, the higher the fiber content. This is consistent with the aforementioned results using the fiber extraction method and density measurements along the flow path. This increase in fiber content along the flow length can probably be explained by the fiber demixing in the layers solidified during filling at high shear rates. Nevertheless, depending on the distance from the injection gate, the content of the fiber clusters increased. Due to their low aspect ratio, this type of fiber is more easily carried by the flow during the mold filling.

Regarding the in-thickness fiber orientation in greater detail, a shell/core structure is clearly visible for the samples taken at the cavity entry (Figure 10a) and in the near-end-of-fill region (Figure 10c). Both zones are subjected to elongational flows. The reduced core layer in Figure 10a shows the impact of packing pressure near the injection gate. For the lubrication region, the orientation state has a flat profile and is almost isotropic with a very high component in the thickness direction (Figure 10b). We can assume that the flow is less elongational in this region compared to the cavity entry and near-end-of-fill region, thus inhibiting the building of the shell/core structure.

### 3.3. Anisotropy Using Ultrasonic Measurements

To assess the anisotropy observed in the fiber orientation measurement by analyzing the µ-tomography scans, experiments with ultrasonic waves were performed. Here, shear wave velocity in neat PP and biocomposite samples are compared to evaluate the increase in wave velocity induced by hemp fibers.

**Figure 11:**
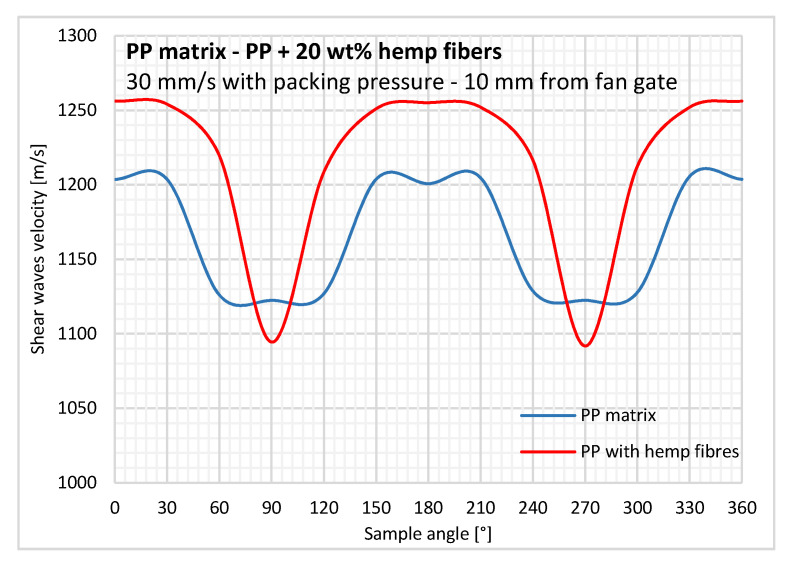
Shear wave velocity changes over a 360° rotation for the neat PP matrix and PP reinforced with hemp fibers.

A non-negligible orientation of the neat PP sample in the injection direction (i.e., 0 and 180°) is characterized by ultrasonic measurements, with a profile close to that obtained for the unidirectional material (Figure 11). The average acoustic birefringence, which indicates the degree of anisotropy in the sample with a main direction, is evaluated at 7%. This preferential orientation is caused by shear-induced crystallization, which develops a specific oriented microstructure in the longitudinal direction. For this reason, the PP matrix should not be considered to be isotropic for anisotropy analysis in the composite material.

For PP reinforced with hemp fibers (Figure 11), very low wave velocities were obtained around the transverse (cross-flow) direction (i.e., 90 and 270°). For all other angles, shear wave velocity was higher with hemp fibers, with a maximum in the longitudinal direction (i.e., 0 and 180°). The average acoustic birefringence was evaluated at 13%, as velocity in the transverse direction is lower than with neat PP.

Unexpectedly, the wave velocities measured at angles around 90 and 270° for the biocomposite are lower than those measured in the neat PP matrix. The addition of reinforcing hemp fibers, even when oriented slightly in this direction, should increase the velocity. As the propagation speed of transverse waves in a medium is proportional to the shear modulus, a decrease in the wave velocity in the transverse direction would mean a loss of rigidity due to the hemp fibers. There are two possible explanations for these results: The hemp fibers may be mechanically anisotropic with a very low transverse modulus or an ultrasonic impedance mismatch may exist at the fiber/matrix interfaces.

For the deduction of the velocity increase induced by the hemp fibers, the shear wave velocity was compared in the neat PP matrix and biocomposite. Thus, for each angle analyzed, the velocity gap induced by the fibers is calculated by considering the biocomposite to be homogeneous. These gaps are then used to calculate the first two components of the second-order orientation tensor using the following formulas:

(1) $$a_{11}=\overset{\pi }{\underset{i=0}{\sum }}V_{SW}\left(i\right)\cos^{2}i$$

(2) $$a_{22}=\overset{\pi }{\underset{i=0}{\sum }}V_{SW}\left(i\right)\sin^{2}i$$

where *i* is the angle to the injection direction of the analyzed sample, ranging from 0 to 180°, and *V_SW_(i)* is the velocity gap induced by the hemp fibers for the corresponding *i* angle.

**Figure 12:**
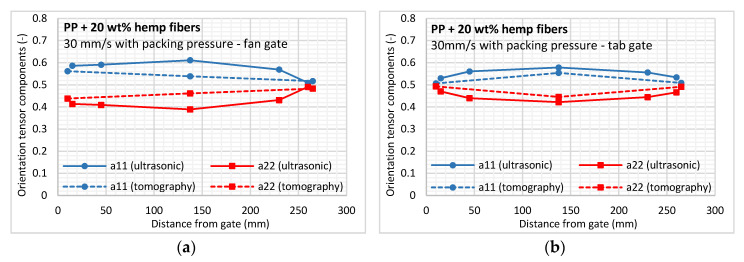
In-plane fiber orientation components from ultrasonic and tomography methods obtained for the (**a**) fan gate and (**b**) tab gate configurations.

Regarding the orientation results obtained from the ultrasonic measurements, a substantial effect of the feeding system (or gate configuration) is visible at the mold cavity entry (up to 50 mm from the gate) (Figure 12). For the fan gate, the orientation in the flow direction (a_11_) is logically higher than the orientation obtained for the tab gate, because a dominant extensional flow is induced by the radial flow front. In the middle of the cavity, results from both gate configurations are comparable (a_11_ = 0.6; a_22_ = 0.4), pointing to a similar material flow in the cavity. Lastly, in the near-end-of-fill region, the orientation state in the flow direction begins to decrease due to the mold edge effects and elongational flow at the flow front.

Due to the possible mechanical anisotropic behavior of the hemp fibers, the calculated values of the orientation tensor components should be considered cautiously, as should those obtained from X-ray tomography, because natural fibers like hemp exhibit a high dispersion in the fiber dimensions. Thus, the scan resolution (i.e., 5.45 μm) may be too coarse to distinguish single μ-fibrils with diameters of 5 μm or less. Due to their shape, these fibers are most likely to be oriented along the injection direction under high shear rates, possibly explaining the underestimation observed when comparing the global anisotropy with ultrasonic measurements.

### 3.4. Elastic Mechanical Properties

#### 3.4.1. Young’s Modulus Characterization

**Figure 13:**
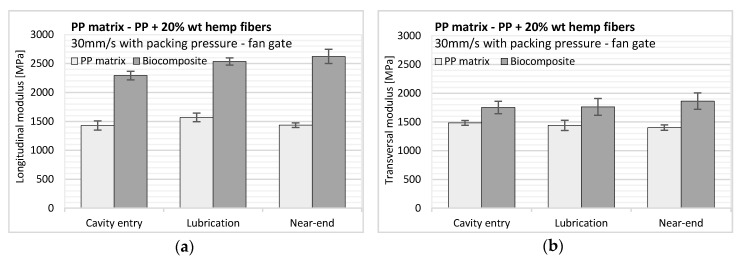
Tensile Young’s modulus versus sample position for the neat PP matrix (left bars) and PP reinforced with hemp fibers (right bars) in (**a**) longitudinal and (**b**) transverse directions

The average modulus of the neat PP matrix is quite high for a neat PP (Figure 13): 1480 MPa and 1440 MPa in longitudinal and transverse directions, respectively. The longitudinal modulus is about 2% higher than the transverse modulus. This shows that the molecular orientation initially characterized by ultrasonic measurements has not a significant impact on resultant elastic properties. The standard deviation in modulus, obtained for 20 measurements, is around 80 MPa. Thus, no variation of crystallinity with the flow length was suspected. The maximum modulus is found in the lubrication region where the molecular orientation is supposed to be the most significant during filling. The tensile strength varies from 23 to 27 MPa in accordance with supplier data, and the PP matrix adopts a more fragile behavior in transverse direction with an elongation at break of 4%. In longitudinal direction, the elongation at break was at least twice as high than in transverse direction with a large dispersion and sometimes values as high as 20%.

For the biocomposite samples, the greater the distance from the injection gate, the higher the moduli (Figure 13). When comparing samples located in the entry and near-end-of-fill regions, we observe an increase in tensile Young’s modulus of 14% and 6.3% in longitudinal and transverse directions, respectively. This result confirms that the fiber content in the samples increases with the flow length; otherwise an increase in the modulus in one direction would inevitably imply a decrease in the perpendicular direction.

Despite increasing the overall stiffness of the material, hemp fibers do not impact the elongation at break in a consequent way in both directions compared to neat PP. The mechanical behavior in longitudinal direction is more fragile and the elongation at break is not impacted by hemp fibers in transverse direction and remains close to 4%.

Young’s modulus values, anisotropy, and heterogeneity are higher here than those observed in an industrial situation. In our study, the samples produced using a laboratory mold and the injection conditions (i.e., plasticizing, counter pressure, and gate configuration) are different than the ones for manufactured samples. Such injection conditions promote these heterogeneities and are very helpful to develop good physical models to improve warpage prediction.

#### 3.4.2. Are Hemp Fibers Mechanically Isotropic?

**Figure 14:**
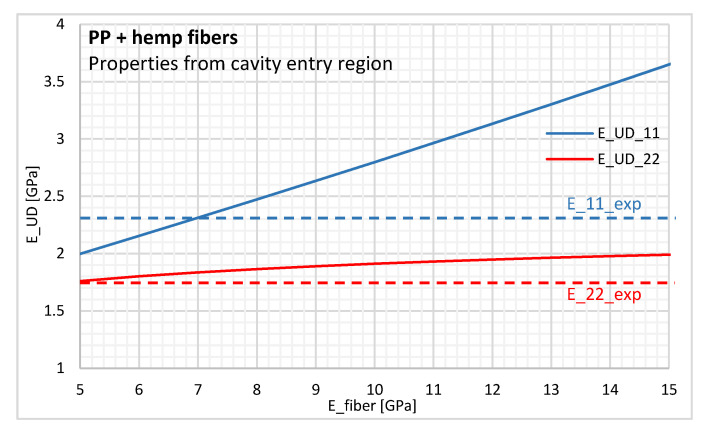
Young’s modulus of the unidirectional composite from the Tandon-Weng model with properties from the cavity entry region.

We propose using the micromechanical Tandon-Weng model [42], equivalent to Mori-Tanaka’s theories of inclusions, while assuming that both materials are isotropic. This model enables us to assess the effective elastic moduli of a transversely isotropic composite with unidirectionally aligned short fibers. We varied the hemp fiber modulus to calculate the homogeneous modulus of the unidirectional composite (Figure 14) with the data provided in Table 2 for the cavity entry.

In the fiber modulus range imposed, the elastic modulus calculated with the micromechanical model would never be representative of the experimental composite Young’s modulus, because the unidirectionally aligned short fibers (E_UD_22) is expected to be lower than the modulus of injected composite determined in the transverse direction (E_22_exp). Only the strong anisotropic behavior of the hemp fibers could explain this result. As both materials are mechanically anisotropic, we do not explore this very complex micromechanics modeling, especially since it is beyond the scope of this paper. Due to the low fiber orientation in the biocomposite, we can only infer that hemp fibers show a significant degree of anisotropy in the mechanical properties, with Young’s modulus being above 8 GPa in the longitudinal direction and below 5 GPa in the transverse direction.

The literature does not provide consistent information on the elastic properties of hemp fibers due to their high variability. Several authors recorded Young’s modulus for hemp fibers in the range of 30 to 70 GPa, without giving information about the dimensions or composition of the fibers studied [5,6,7]. Only a few studies mention the anisotropic behavior of natural fibers like hemp [43,44]. The authors attempted to estimate the transverse thermoelastic properties of natural fibers through a combination of experimental measurements and micromechanical modeling on unidirectionally aligned short fiber composites. Their results showed considerable anisotropy in the elastic properties of natural fibers like jute, flax, and sisal. The degree of anisotropy depends on the fiber type, although it is essentially linked to cellulose content and internal microstructure. Bourmaud et al. obtained a transverse modulus of 5 ± 1.5 GPa for hemp fibers using the nanoindentation technique [45].

### 3.5. CTE Anisotropy Measurement

**Figure 15:**
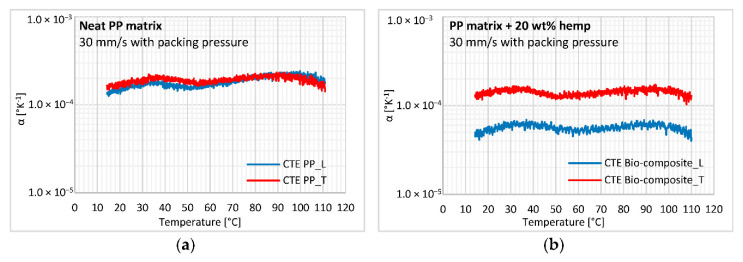
Changes in longitudinal and transverse coefficient of thermal expansion (CTE) during cooling for the (**a**) neat PP matrix and (**b**) PP reinforced with hemp fibers.

The linear coefficient of thermal expansion (CTE) for samples cut in longitudinal and transverse directions is measured for the neat PP matrix and biocomposite in cooling mode (Figure 15). The CTE measured during heating leads to a non-linear variation in CTE, with a significant residual deformation of about 1% for neat PP due to the relaxation of stresses induced by cooling. First, we can observe that the CTE for both materials remains constant over the temperature range considered (i.e., 10 to 110 °C). For the neat matrix, no significant anisotropy of CTE was found with a nearly constant value of 1.75 × 10^−4^ K^−1^ (Figure 15a). The crystalline orientation, mechanically characterized using ultrasonic and tensile test measurements, does not impact the thermal behavior of neat PP. For the biocomposite, a constant anisotropy is characterized over the temperature range (Figure 15b). Regarding the results for neat PP, we can infer that this anisotropy is only due to the addition of hemp fibers and their orientation induced by the process. These oriented fibers induce anisotropic shrinkage that occurs with cooling during the process and leads to part warpage [46].

Comparing CTE values from the neat PP matrix and biocomposite, we can first observe that the CTE of the biocomposite in the transverse direction is very close to that of neat PP, while it is 2.5 times lower in the longitudinal direction. Considering the orientation state discussed previously, this result is unpredictable in micromechanical models when assuming that both materials are isotropic. As for mechanical testing, the CTE experiments highlight the strong anisotropy in the elastic properties of hemp fibers. Some authors combine experimental measurements and micromechanical modeling on unidirectionally aligned short fiber composites to assess the CTE of natural fibers in longitudinal and transverse directions [43,44]. For all the studied fibers, a negative longitudinal CTE was shown for temperatures ranging from −50 to 50 °C, about −1.0 × 10^−5^ K^−1^ at room temperature, whereas the transverse CTE was close to that of the polymeric matrix, namely 8.0 × 10^−5^ K^−1^.

### 3.6. Shrinkage Measurements

Shrinkage in injection molding is usually defined for the injection (α∥) and cross-flow directions (α⊥). To measure them, small markers were placed on the mold surface at strategic positions.

**Figure 16:**
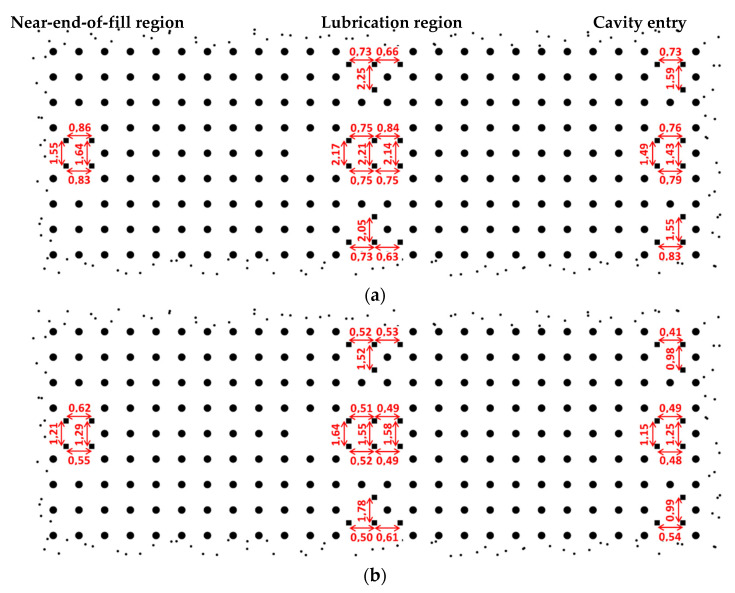
Longitudinal and transverse shrinkage measurements (in %) on biocomposite parts injected at 30 mm/s (**a**) without and (**b**) with packing pressure.

Regarding Figure 16, a consequent anisotropy of shrinkage is observed as the one of mechanical measurements and preferential orientation of fibers in the injection direction. The variation of α∥ along the flow length is extremely limited for both injection conditions, whereas it is greater for α⊥. Shrinkage is significantly reduced in both directions when packing pressure is applied (Figure 16b). However, except for the cavity entry where material flow is highly affected by gate configuration, the anisotropy ratio α∥/α⊥ is equivalent to that measured on the part without packing pressure (Figure 16a). This anisotropy of shrinkage in the fiber-reinforced material mainly follows that of CTE. Lastly, a slight decrease in α⊥ occurs in the near-end-of-fill region due to the non-erasure of the fountain flow effect. The fiber content measured here, which plays a role in shrinkage phenomenon, was also much higher than the rest of the part. Lastly, the edge part, which had a higher cooling rate due to the additional mold wall, had no influence on α∥.

Part shrinkage in injection molding can be linked to thermal elastic properties as well as stress relaxation during the process cooling [22]. This stress relaxation is visible in the first heating stage of the dilatometric measurement, leading to a residual deformation of the sample at the end of the test after returning to the initial temperature. To reproduce such behavior in simulation codes, a viscoelastic constitutive model of the material should be implemented.

## 4. Conclusions

PP reinforced by hemp becomes widely used for parts, especially for interior automotive parts processed by injection molding, but simulation codes fail to predict accurate thermomechanical properties, so shrinkage and part deformation. Therefore, the present work aimed to characterize the induced microstructure of injection-molded PP reinforced by hemp fibers and its influence on thermomechanical properties.

In-thickness fiber orientation and fiber content were determined by X-ray tomography along the flow. A typical shell/core structure was observed for the fiber orientation in injection molding, but it was much less pronounced than that found in glass and carbon fibers. Moreover, a high fiber orientation in the thickness direction was identified, ranging from 0.1 to 0.15, which is at least twice as high as glass fibers. It is supposed that the flexibility, high aspect ratio, and high degree of tortuosity of hemp fibers reduce their orientation ability by significantly increasing the fiber interaction probability.

Considering in-thickness fiber content, we identified two symmetrical layers with a very low fiber content (<10% in weight) and a paradoxical isotropic fiber orientation state. They correspond to the layers solidified during mold filling and thus subjected to very high shear rates. The fibers are demixed and probably carried further in the flow, which is shown by a linear increase along the flow path of about 2%/100 mm of shear flow, determined by density measurements and by direct fiber content measurements taken after matrix dissolution.

The impact of the feeding system on material microstructure was shown to be limited up to 50 mm from the injection gate. The tab gate generates an elongational flow at the cavity entry, characterized by a higher cross-flow orientation using X-ray tomography and ultrasonic measurements. The packing pressure applied after mold filling was shown to reduce the effect of elongational flow, thus orienting fibers in the central layer.

In this work, a high degree of anisotropy was observed for the biocomposite in the tensile tests and CTE measurements. This thermomechanical anisotropy was, however, unpredictable when using simple micromechanical models given the mechanically isotropic constituents. This result highlights the significant anisotropy in the thermoelastic properties of hemp fibers.

Due to their high flexibility and tortuosity, hemp fibers reveal a specific thickness and spatial distribution, which should be considered in simulation codes to accurately predict thermoelastic properties, shrinkage, and warpage, by introducing more diffusion in orientation model than the one used for glass fiber. As a minimum, the introduction of fiber content depending on flow length should be added based on what is already known about particle migration. Lastly, the anisotropic thermoelastic behavior of the fibers should be taken into account in the numerical analysis to improve the prediction of part shrinkage and warpage in order to better design molds.

## Figures and Tables

**Table 1:** Injection conditions used for fiber orientation characterization in the study.

Injection Configuration	Injection Ram Speed [mm/s]/Injection Time [s]	Packing Pressure [MPa]
Fan gate/Tab gate	30–45/2.4–1.6	0–40

**Table 2:** Input parameters used to assess unidirectionally aligned short fiber moduli.

Position	Cavity Entry
E_PP_ [GPa]	1.45
ν_PP_	0.41
E_fiber_ [GPa]	Varying from 5 to 15
ν_fiber_	0.20
Fiber aspect ratio	20
Fiber volumetric content [–]	0.1482
